# Supplementary material for: Seroprevalence of anti-SARS-CoV-2 antibodies before and after implementation of anti-COVID-19 vaccination among hospital staff in Bangui, Central African Republic
Source: PLOS Glob Public Health. 2023 Nov 1;3(11):e0001497. doi: 10.1371/journal.pgph.0001497 (PMC10619860; doi:10.1371/journal.pgph.0001497)
Supplement: Attachment — Submitted filename: Response to reviewers.docx [file pgph.0001497.s002.docx]

Alexandre Manirakiza	Bangui, 01 July 2023

Institut Pasteur of Bangui,
International Network of Instituts Pasteur
PO box 923, Pasteur Avenue, Bangui
Central African Republic

e-mail : amanirak@yahoo.fr

Phone : +236 72 10 79 16

Dear Reviiewers,

Thank you for the comments and remarks provided to us on our manuscript PGPH-D-22-02038, entitled : “Seroprevalence of anti-SARS-CoV-2 antibodies before and after implementation of anti-COVID-19 vaccination among hospital staff in Bangui, Central African Republic »

We have revised the manuscript accordingly. The responses are detailed as follows.

Regards,

Alexandre Manirakiza

PGPH-D-22-02038
Seroprevalence of anti-SARS-CoV-2 antibodies before and after implementation of anti-COVID-19 vaccination among hospital staff in Bangui, Central African Republic
PLOS Global Public Health

Dear Dr. Alezandre Manirakiza

Thank you for submitting your manuscript to PLOS Global Public Health. After careful consideration, we feel that it has merit but does not fully meet PLOS Global Public Health’s publication criteria as it currently stands. Therefore, we invite you to submit a revised version of the manuscript that addresses the points raised during the review process.

The reviewers have added many comments intended to improve the paper. I look forward to reading the final version as this is important work and we will hope to publish it in its future form.

Do specify which antibody tests were used. There are S (Spike protein) and N(nucleocapsid) tests; the former picks up vaccination and infection immunity, the latter only infected-based immunity.

**Authors’ response** : we stated in the manuscript that «  For blood samples, the miniVidas was used for assessing vidas SARS-CoV-2 IgM and IgG (Spike protein) assays by enzyme-linked fluorescent assay (ELFA).”

Do we have a sense of the timing of the vaccination between the two surveys, since immunity can wane with time?

**Authors’ response**  : The vaccination campain was carried from May 2021. We stated the fact that immunity cane wane with time as a limitation on four study as follows : « Another limitation of our study is that we did not evaluate antibody titers. Indeed, according to studies, IgG levels are waning rapidly by weeks after infection/vaccination or reamain at stable levels over months. hence a need to define the specific antibody titers that correlate of protection ».

Please clarify the vaccination dosages given more clearly on lines 143-5: "*the majority (82, or 70.8%) had received the Astra Zeneca vaccine, followed by the Johnson & Johnson vaccine (30, or 25.8%), and 4 (3.4%) had received a single dose of any of these two type of the COVID-19 vaccine."* J&J is usually single dose so do clarify that 82 had 2 doses of AZ, 30 had 1 dose J&J, and 4 had one dose of a two dose vaccine, if that is correct.

**Authors’ response** : These details had been added to the manuscript as follows : « Among HCWs who received Astra Zeneca vaccine, 87.8% had 2 doses, and a 90.0% of HCWs who received Johnson & Johnson vaccine had 2 doses.”

**Reviewer #2: 1. Methods section**
• Four (4) health facilities were sampled in the first and second surveys. However, two of the facilities are not consistent between the first and the second survey. This means that for the second survey, the population sampled for these two facilities is different from the first survey hence not “repeated” as the methodology mentions. It is not reported as to why there was a change and how it impacted analysis of the data.

**Authors’ response** : We added in the manuscript that « The second survey was conducted in May 2022 at the University Hospital of the Sino-Central African Friendship (CHUASC) and the Community University Hospital (CHUC) and the Centre Hospitalier Universitaire Maman Elisabeth Domitien (CHUMED) as CHUPB HCWs did not comply again for their participation in the survey due to some internal admnistrative concerns and some of them were transferred in CHUMED”.

A brief description of the health facilities would be useful. Capacity, category (such as regional, referral, super-specialised or otherwise etc.)

**Authors’ response** : A description of the health facilities was added as follows : CHUPB, CHUMED, CHUASC and CHUC are national reference teaching hospitals located in Bangui, the capital of CAR. The surface area of Bangui is 67 km2 and has a population of 1,145,280, with a density of 17,094 inhabitants per km2. The main missions of these hospitals are preventive and promotional care, curative care, training and research. Each of these hospitals has emergency, outpatient and inpatient departments. The number of healthcare staff are 407, 150 and 413 and 540, while their bed capacities are 296, 100, 300 and 800, respectively. Moreover, there is regular mobility of healthcare staff, particularly nurses and midwives, between these university hospitals. The Figure 1 provide the locations of these hospitals

• Lines 121-123: ….. The nasopharyngeal swabs were used for viral detection using RT-PCR (retro-transcriptase polymerase chain reaction) (polymerase chain reaction). Mention which virus is being referred to for viral detection, remove the second bracket and its words which are repetition and for the term “retro-transcriptase” I believe the author meant “reverse-transcriptase”.

**Authors’ response**  : Thank you, This change was made. It is reverse-transcriptase polymerase chain reaction instead of retro-transcriptase polymerase chain ; and we removed the second bracket and its words

• Occupation: In the manuscript, the breakdown of occupation is limited to medical doctors, nurses and midwives. Other cadres such as radiologists, lab, surgeons are important as well to define due to close contact with patients and samples.

**Authors’ response** : Thank you. We reviewed the breakdown of the occupation and considered « Close contact staff with patients » and « Others ». « Close contact staff with patients » were medical doctors, nurses, midwives, radiologists, laboratory technicians and surgeons statf, and we stated in the manuscript that « Close contact staff with patients were more likely to have a positive serological status (IgM and/or IgG) against SARS-CoV-2 compared with others staffs ([1.3-4.3]; P = 0.005)”

• Line 125-126: To compare the two-sampling time points, the sampling should have been comparable, however, for the second survey, only blood samples were collected, while for the first survey, both blood and nasopharyngeal swabs were collected. Did the study test for positive SARS-CoV-2 samples in the second survey? At least for IgM positive?

**Authors’ response** : Thank you for the remarks. As this study aimed to « evaluate any changes in the anti-COVID-19 serological status before and after the vaccination campaign of HCWs » we hawe modified accordingly the text by delating the phrase in the « method «  section: « During the first survey, nasopharyngeal swabs were collected and a blood sample of approximately 1 to 2 mL was taken in a dry tube. These samples were then sent to the Institut Pasteur of Bangui for laboratory analysis. The nasopharyngeal swabs collected during the were used for viral detection using RT-PCR (reverse-transcriptase polymerase chain reaction)” and related results in the Table 1.

• Which ELISA kit/ brand was used?

**Authors’ response** : This precision was made in the manuscript as follows : « During the first and second surveys, we collected demographic data (sex and age) and qualification of the participating HCWs and blood sample of approximately 1 to 2 mL was taken in a dry tube and sent to the Institut Pasteur of Bangui where the miniVidas was used for assessing SARS-CoV-2 IgM and IgG (Spike protein by enzyme-linked fluorescent assay (ELFA)”.

• Line 144-145: states that 4 (3.4%) had received a single dose of any of the two types of COVID vaccine (AstraZeneca and J&J). To note is that the J&J vaccine was a single-dose vaccine.

**Authors’ response** : We stated in the manuscript that «Among HCWs who received Astra Zeneca vaccine, 87.8% had 2 doses, and a 90.0% of HCWs who received Johnson & Johnson vaccine had 2 doses”

2. Results
• The two health facilities that were sampled only in the first survey or only in the second survey. These cannot be analysed within the scope of this manuscript, and the authors should consider omitting them from this manuscript. The author should only maintain the health facilities that were sampled at both time points.

**Authors’ response** : Thank for the reamarks. We stated in the manuscript that « The second survey was conducted in May 2022 at the University Hospital of the Sino-Central African Friendship (CHUASC) and the Community University Hospital (CHUC) and the Centre Hospitalier Universitaire Maman Elisabeth Domitien (CHUMED) as CHUPB HCWs did not comply again for their participation in the survey due to some internal administrative concerns and some of them were transferred in CHUMED” .

• Table 1:
i. The ratio of male/female. The authors have included a p-value. The ratio of male/female would reflect the sex ratio of staff at their workplace therefore, no analysis between the two sexes can be done.

**Authors’ response** : Thank you. This change was made.

ii. Occupation: A more detailed breakdown is needed. Example diagnostic staff such as laboratory staff, radiologists etc. These cadres have close access to patients and samples.

**Authors’ response** : Details are made in table 1. Indeed, we put medical doctors, nurses, midwives, radiologists, laboratory technicians and surgeons statf in a same category as « Close contact staff with patients »

iii. What was the reason for not performing a SARS-CoV-2 PCR test during the second survey? All should have been tested for the virus as per the first survey. Gives an indication of infection during the second survey, re-infection, onset and duration of IgM/IgG.

**Authors’ response** : Thank you. As the objective of this study was to « evaluate any changes in the anti-COVID-19 serological status before and after the vaccination campaign of HCWs” we focused on this aspect. We delated the results of SARS-CoV-2 PCR test during the first survey.

iv. A useful addition to the information in this manuscript is the antibody titres seen in both surveys and between vaccines.

**Authors’ response** : Thank you. This limit was presented in the discussion .

v. If SARS-CoV-2 results are available for the 5 who tested IgM-positive in the second survey, this information should be in the manuscript. This may give an

**Authors’ response** : As this study aimed to « evaluate any changes in the anti-COVID-19 serological status before and after the vaccination campaign of HCWs » we hawe modified accordingly the text by delating the phrase in the « method «  section: « During the first survey, nasopharyngeal swabs were collected and a blood sample of approximately 1 to 2 mL was taken in a dry tube. These samples were then sent to the Institut Pasteur of Bangui for laboratory analysis. The nasopharyngeal swabs collected during the were used for viral detection using RT-PCR (reverse-transcriptase polymerase chain reaction)” and related results in the Table 1.

So, in the second survey, we did not perfom PCR, to identify SARS-CoV-2

vi. Did any of the HCWs have both IgG and IgM?

**Authors’ response**  : Thank you. We stated in the manuscript that « Nine HCWs have both IgG and IgM in the first survey and four HCWs have both IgG and IgM in the second one ».

vii. Line 160-161: What does it mean when you say you conducted the survey successfully in two facilities i.e CHUC and CHUASC? What went wrong with the other two CHUMED and CHUPB?

**Authors’ response** : We added in the manuscript that « The second survey was conducted in May 2022 at the University Hospital of the Sino-Central African Friendship (CHUASC) and the Community University Hospital (CHUC) and the Centre Hospitalier Universitaire Maman Elisabeth Domitien (CHUMED) as CHUPB HCWs did not comply again for their participation in the survey due to some internal admnistrative concerns and some of them were transferred in CHUMED”. This have been stated in the limitations of this study.

viii. Which vaccine elicited the highest titre levels? AZ or J&J?

**Authors’ response** : Thank your, remark. The serological analysis in this study was based on the miniVidas for assessing SARS-CoV-2 IgM and IgG (Spike protein by enzyme-linked fluorescent assay (ELFA)”. However, we did not assess the the titre level related to vaccines.

3. Discussion
• Very brief. Expand on the discussion. Discuss the results with findings from other settings.

**Authors’ response** : Thank you. Was taken into account in the discussion.

• Discuss the significant results.

**Authors’ response** : Thank you. These changes have been made.

• Limitations of the study should be mentioned e.g. testing limitations that may lead to false positive or negative results. Limitations of the methodology used.

**Authors’ response** : Thank you, limitations of this study was added to the manuscript. However, in the methodology section, we stated that «the performance of this test has been previously evaluated, as a internal control of quality at Institut Pasteur of Bangui, using 180 plasma bank samples from before May 2019, resulting in a specificity of 99·6%”.

4. Conclusion
• There is a general comment in the conclusion that states that all HCWs in Bangui developed IgG antibodies by May 2021. The value of 95.7% is not 100% there are some negatives. Also, the authors did not indicate whether the facilities mentioned in Bangui are all of them.
**Authors’ response** : Thank you. We have made this more clear in stating that « Our findings showed a sharp increase prevalence of IgG antibodies to SARS-CoV-2 by May 2022 compared to that of one year before”

5. References
• General comment for all references: Use the correct reference format for this journal “Vancouver”.
• Reference 12: At the end, it is stated that it was accepted for publication. If so, cite the accepted version with the DOI. I could not find it online.
• Reference 17: It is a Medriv reference. Reference the actual peer-reviewed publication.

**Authors’ response** : These changes have been made.

Reviewer #3: congratulation in your study

please find some of the inputs on the research report to work on
1.please align the the report in a presentable manner where one can easily find the required information(Headings and sub- headings)

**Authors’ response** : These changes have been made.

2. you can only use one format of data presentation either percentages, ratios or exact number to avoid bulk and similar data that may lead to confusion

**Authors’ response** : These changes have been made.
