# Supplementary material for: Seroprevalence of anti-SARS-CoV-2 antibodies before and after implementation of anti-COVID-19 vaccination among hospital staff in Bangui, Central African Republic
Source: PLOS Glob Public Health. 2023 Nov 1;3(11):e0001497. doi: 10.1371/journal.pgph.0001497 (PMC10619860; doi:10.1371/journal.pgph.0001497)
Supplement: Attachment — Submitted filename: Response to Reviews2.docx [file pgph.0001497.s003.docx]

**PGPH-D-22-02038R2**
Seroprevalence of anti-SARS-CoV-2 antibodies before and after implementation of anti-COVID-19 vaccination among hospital staff in Bangui, Central African Republic
PLOS Global Public Health

Dear Dr. Manirakiza: 

Thank you for submitting your manuscript to PLOS Global Public Health. After careful consideration, we feel that it has merit but does not fully meet PLOS Global Public Health’s publication criteria as it currently stands. Therefore, we invite you to submit a revised version of the manuscript that addresses the points raised during the review process.

The paper is almost ready for publication. Please reference the reviewer comments shared. In particular, given the difference in significance with occupation there should be more discussion and explanation as it can be confusing. originally, doctor, nurse/midwife, and other was not significant but is now that it is close contact vs support staff, there should be more discussion of this point and also more clarification of what close contact is. I would include a table of what close contacts are considered, as this is not a standardized term and the meaning may be inferred differently. In fact, radiologist may not be considered by many to have close patient contact, but a radiology tech would; however, in different settings the roles may vary and this should be clarified.

**Authors’ response :** We have stated in the Method section that « Included were all staff administering health care to patients (medical doctors, nurses, midwives, radiology technicians, laboratory technicians and surgeons staff) and support staff (health personel having non-clinical functions) in the healthcare departments in the hospitals. Hence, this staff administering health care to patients was considered as close contact staff because they spent a lot of time with patients presenting various diseases whose infectious status to COVID-19 is unknown and most of the time they do not wear appropriate personal protective equipment”.

Moreover, I would also provide more discussion on the N (nucleocapsid) vs S (spike) IgG testing, as here only S available, which is a limitation as cannot include the knowledge N tests would have added to the S data (ie as N positive only after infection, while S positive after both vaccination and infection).

**Authors’ response :** We have stated in the Discussion section that : « During the same period of this survey in general population, a vaccination campaign against COVID-19 was carried out among healthcare staff. This underscores that the HCW surveyed in our study, less than one year after this campaign, have hybrid immunity (SARS-CoV-2 infection combined with anti-COVID-19 vaccination). However, the detection of anti-S antibodies did not allow us to differentiate between vaccinated individuals and others with or without a history of infection, which would have been possible if we had detected anti-nucleocapsid (anti-N) antibodies. However, it have been showed that anti-S protein and anti-N persisted up to 12 months after infection, but 97% of individuals retained their anti-S while only 20% retained their anti-N antibodies (20). Furthermore, prior SARS-CoV-2 infection increases the titers of SARS-CoV-2 anti-S antibodies responses elicited by subsequent vaccination (21).

We look forward to receiving your revised manuscript.

Kind regards,

Megan Coffee, MD, PhD
Academic Editor
PLOS Global Public Health

Journal Requirements

Please review your reference list to ensure that it is complete and correct. If you have cited papers that have been retracted, please include the rationale for doing so in the manuscript text, or remove these references and replace them with relevant current references. Any changes to the reference list should be mentioned in the rebuttal letter that accompanies your revised manuscript. If you need to cite a retracted article, indicate the article’s retracted status in the References list and also include a citation and full reference for the retraction notice.

Additional Editor Comments (if provided):
[Note: HTML markup is below. Please do not edit.]

Reviewers' comments:

Reviewer's Responses to Questions

**Comments to the Author**

1. If the authors have adequately addressed your comments raised in a previous round of review and you feel that this manuscript is now acceptable for publication, you may indicate that here to bypass the “Comments to the Author” section, enter your conflict of interest statement in the “Confidential to Editor” section, and submit your "Accept" recommendation.

Reviewer #2: (No Response)

Reviewer #3: All comments have been addressed

2. Does this manuscript meet PLOS Global Public Health’s [publication criteria](https://journals.plos.org/globalpublichealth/s/criteria-for-publication)? Is the manuscript technically sound, and do the data support the conclusions? The manuscript must describe methodologically and ethically rigorous research with conclusions that are appropriately drawn based on the data presented.

Reviewer #2: Partly

Reviewer #3: Yes

3. Has the statistical analysis been performed appropriately and rigorously?

Reviewer #2: I don't know

Reviewer #3: Yes

4. Have the authors made all data underlying the findings in their manuscript fully available (please refer to the Data Availability Statement at the start of the manuscript PDF file)?

The [PLOS Data policy](https://journals.plos.org/globalpublichealth/s/data-availability) requires authors to make all data underlying the findings described in their manuscript fully available without restriction, with rare exception. The data should be provided as part of the manuscript or its supporting information, or deposited to a public repository. For example, in addition to summary statistics, the data points behind means, medians and variance measures should be available. If there are restrictions on publicly sharing data—e.g. participant privacy or use of data from a third party—those must be specified.

Reviewer #2: No

Reviewer #3: Yes

5. Is the manuscript presented in an intelligible fashion and written in standard English?

PLOS Global Public Health does not copyedit accepted manuscripts, so the language in submitted articles must be clear, correct, and unambiguous. Any typographical or grammatical errors should be corrected at revision, so please note any specific errors here.

Reviewer #2: No

Reviewer #3: Yes

6. Review Comments to the Author

Please use the space provided to explain your answers to the questions above. You may also include additional comments for the author, including concerns about dual publication, research ethics, or publication ethics. (Please upload your review as an attachment if it exceeds 20,000 characters)

Reviewer #2: The authors have attempted to respond to all comments by the authors however there are still some clarifications that are still needed as follows:
1. General comment: Check for typographical and grammatical errors specifically for the newly added text e.g. Line 163: word “statf” change to “staff”.

**Authors’ response** : This change was made.

2. Line 94: The author has stated that CHUPS HCW did not “comply” again for their participation in the survey. I suggest that the word “comply” is changed to “consent”. I suggest that the sentence ends at the word “survey” there is no need for further explanation. I suggest removing the part that says, “due to some internal administrative concerns and some of them were transferred at CHUMED”.

**Authors’ response** : This change was made.

3. Line 164: grammar: “Support staff was non-clinical functions” suggest to change the word “was” to “having” or another similar word.

**Authors’ response** : This change was made.

4. Table 1: HCWs occupation: the p-value is now significant. This needs to be clarified in the rebuttal. Data from both time-points should be provided for review.

**Authors’ response** : In the Discussion section, we stated that « Close contacts HCWs with patients had the highest overall seroprevalence. They were more likely to have a positive serology not only because of the anti-COVID-19 vaccination, but also because they are high risk of being infected with SARS-CoV-2 from patients.

5. Discussion section: lines 7-10. Put a reference.

**Authors’ response :** The reference was put.

6. In the rebuttal, the authors have not indicated changes to the references, such as added or removed references.

Reviewer #3: Congratulations for this achievement
I can't understand why is every line numbered in your manuscript
Also you can pass through it to finalize some grammatic errors so as to provide i better research work
All the best in your publications
Thank you

7. PLOS authors have the option to publish the peer review history of their article ([what does this mean?](https://journals.plos.org/globalpublichealth/s/editorial-and-peer-review-process#loc-peer-review-history)). If published, this will include your full peer review and any attached files.


**Do you want your identity to be public for this peer review?** If you choose “no”, your identity will remain anonymous but your review may still be made public.


For information about this choice, including consent withdrawal, please see our [Privacy Policy](https://www.plos.org/privacy-policy).

Reviewer #2: No

Reviewer #3: No
